# Stability Optimization Strategies of Cathode Materials for Aqueous Zinc Ion Batteries: A Mini Review

**DOI:** 10.3389/fchem.2021.828119

**Published:** 2022-01-20

**Authors:** Yi Gan, Cong Wang, Jingying Li, Junjie Zheng, Ziang Wu, Lin Lv, Pei Liang, Houzhao Wan, Jun Zhang, Hao Wang

**Affiliations:** ^1^ School of Microelectronics, Hubei University, Wuhan, China; ^2^ Hubei Yangtze Memory Laboratories, Wuhan, China; ^3^ College of Optical and Electronic Technology, China Jiliang University, Hangzhou, China

**Keywords:** aqueous zinc ion battery, cathode materials, cyclic stability, stability attenuation, optimization

## Abstract

Among the new energy storage devices, aqueous zinc ion batteries (AZIBs) have become the current research hot spot with significant advantages of low cost, high safety, and environmental protection. However, the cycle stability of cathode materials is unsatisfactory, which leads to great obstacles in the practical application of AZIBs. In recent years, a large number of studies have been carried out systematically and deeply around the optimization strategy of cathode material stability of AZIBs. In this review, the factors of cyclic stability attenuation of cathode materials and the strategies of optimizing the stability of cathode materials for AZIBs by vacancy, doping, object modification, and combination engineering were summarized. In addition, the mechanism and applicable material system of relevant optimization strategies were put forward, and finally, the future research direction was proposed in this article.

## Introduction

In response to the global climate crisis, the research of new energy storage devices has been widely focused on expanding the application of renewable energy to replace fossil energy (Tan et al., 2020a; Wang et al., 2020a; Gan et al., 2020; Cai et al., 2021a; Liu et al., 2021a; Cai et al., 2021b; Deng et al., 2021; Zhao et al., 2021). In the field of new energy storage, lithium-ion batteries have been widely used because of their high energy density and wide working voltage (Park et al., 2021; Xia et al., 2021; Feng et al., 2022). However, the scarcity of lithium resources increases the cost of lithium batteries, and the majority of the organic electrolyte used are poisonous or flammable, reducing the safety of lithium batteries (Li et al., 2021a; Du et al., 2021; Hou et al., 2021). Comparatively, zinc metal has the advantages of non-toxic, low cost, and redox potential, which is more suitable for aqueous electrolytes (Yao et al., 2021). Moreover, the high density and multi-charge of zinc render aqueous zinc ion batteries (AZIBs) with excellent energy density, which makes it have great application prospects (Gao et al., 2021). However, the low cycle stability of AZIBs is an inevitable problem. As one of the most core components, cathode materials for the improvement of AZIB performance critically depend on the optimization of stability. The storage mechanism and capacity attenuation of zinc ions in AZIBs system have not been fully clarified. Thus, the latest research progress is necessary to be summarized, which is conducive to providing the following research direction.

Herein, the primary factors causing the performance degradation of cathode materials for AZIBs are summarized, and optimization strategies for the stability of cathode materials are introduced. Finally, according to the optimization strategy introduced in the summary, some problems to be further studied will be put forward, and the subsequent optimization research of stability will be prospected.

## Performance Degradation of Cathode Materials

The strong electrostatic interaction and large steric effect between divalent Zn^2+^ and the main structure of cathode materials in AZIBs lead to poor cyclicity and very slow intercalation kinetics. Meanwhile, the pH, additives, types, and concentrations of zinc salts in the electrolyte will also affect the energy storage characteristics of cathode materials. The attenuation of cathode material performance is mainly divided into the following situations:

Irreversible phase transition: During the charge–discharge process of the battery, Zn^2+^ intercalation, ion/molecule co-intercalation, and conversion reaction are likely to cause irreversible damage to the structure of cathode materials (Chen et al., 2020). For instance, Zn_x_MnO_2_ will be formed when Zn^2+^ is inserted into the space of MnO_2_ with a layered structure, while MnOOH with a tunnel structure will be formed when H^+^ is inserted into the material in solution (Liu et al., 2021b; Ma et al., 2021). This phase transition in varying degrees will destroy part of the original structure, resulting in the attenuation of performance. Moreover, the H^+^ insertion process is usually accompanied by-products [such as Zn_4_SO_4_(OH)_6_·5H_2_O] with the change of pH, which will cause the adhesion of insulation corrosion on the cathode surface and also continuously reduce the electrochemical activity of the cathode (Li et al., 2019).

Cathodic dissolution: The dissolution and diffusion of cathode materials in electrolytes are irreversible to a certain extent, which will cause the instability of the material structure. For example, the Jahn–Teller effect in high-valence manganese-based oxides induces the irreversible transformation of some Mn^3+^ to Mn^2+^ in the process of cathode discharge and then will destroy the main structure of materials (Heo et al., 2021). In addition, for most material systems such as vanadium-based compounds, Prussian blue and analogs, and their structures are not stable in electrolytes, and irreversible dissolution will occur when the cathode is discharged for a long time (Wan and Niu, 2019; Li et al., 2021b).

In conclusion, the performance degradation of cathode materials is not only due to the influence of the electrolyte environment but also related to its own structural characteristics and reaction mechanism. Moreover, according to the research reported at present, the cycle stability of cathode materials can be optimized from four aspects: introduction of vacancy, substitution/gap doping, object modification, and combination engineering.

## Stability Optimizations for Cathode Materials

### Introduction of Vacancy

**FIGURE 1:**
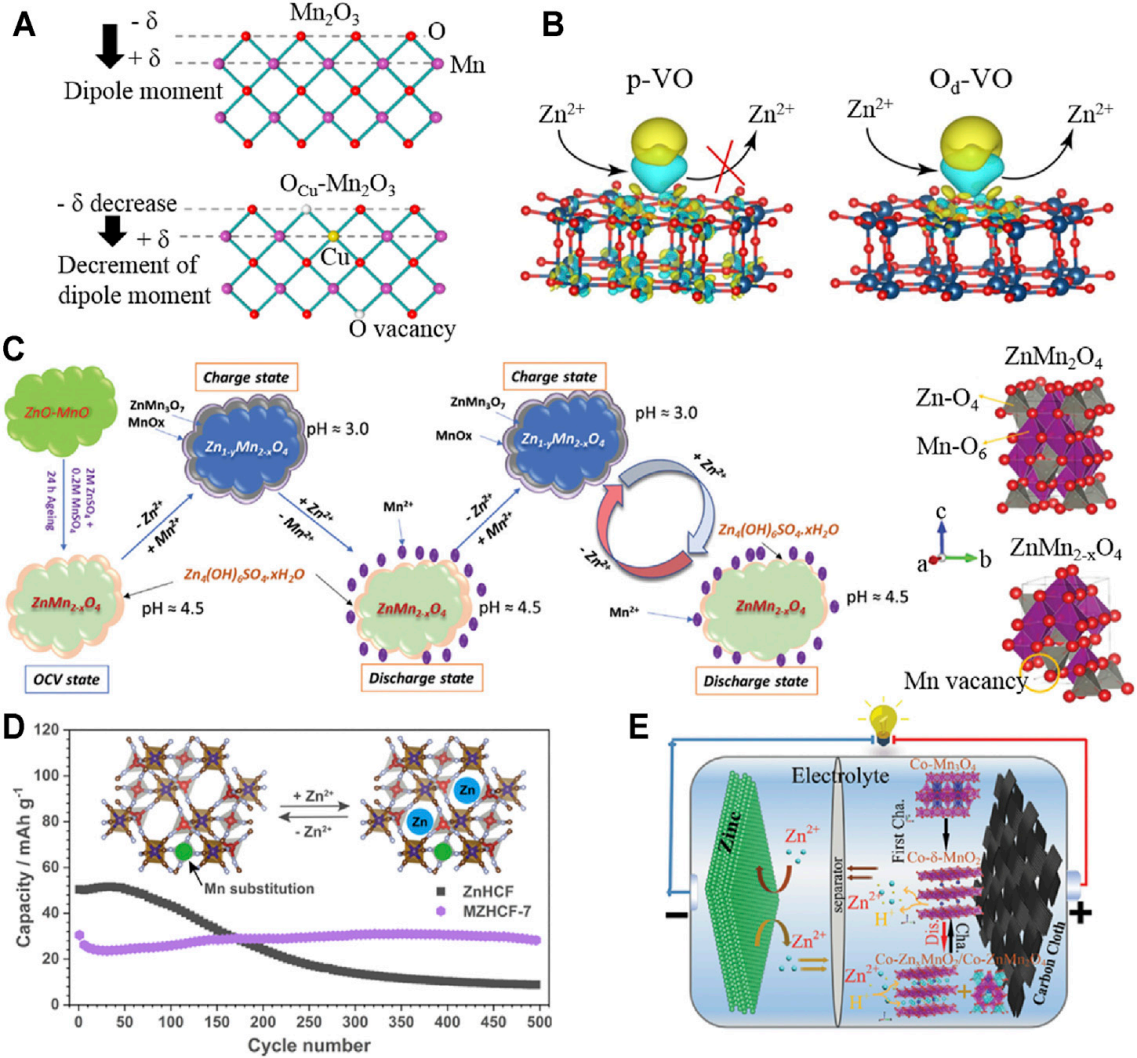
Vacancies and doping modification of cathode materials. **(A)** Atomic structure models of a single layer height in Mn_2_O_3_ and O_cu_-Mn_2_O_3_, respectively; **(B)** illustrations of the Zn^2+^ storage/release for p-VO and O_d_-VO; **(C)** schematic illustration for the reaction mechanism of the *in situ* formed Zn/Mn-d-ZMO@C; **(D)** schematic diagram of the reaction mechanism of MZHCFs; **(E)** schematic illustration of Zn|| Co-Mn_3_O_4_/CAN battery. Reproduced with permission (Liu et al., 2020a; Liao et al., 2020; Islam et al., 2021; Ji et al., 2021; Ni et al., 2021).

The introduction of an appropriate amount of vacancy engineering (oxygen vacancy, metal vacancy, etc.) has been confirmed that it not only can reduce the bandgap, improve the conductivity, and promote the diffusion kinetics of H^+^/Zn^2+^ to improve the capacity but also enhance the structural stability to inhibit its dissolution, so as to improve the cycle stability (Wang et al., 2020b; Luo et al., 2020; Tan et al., 2020b; Cao et al., 2021; Tong et al., 2021; Cui et al., 2022). Zhang et al. achieved the doping of Cu^2+^ substituting Mn^3+^ by solvothermal and annealing and synthesized oxygen-containing vacancy Mn_2_O_3_ (O_Cu_-Mn_2_O_3_) (Liu et al., 2020a). The uniform distribution of oxygen vacancies can adjust the internal electric field and crystal structure by compensating the non-zero dipole moment (in Figure 1A), thereby promoting the reaction kinetics and improving the stability of the crystal structure. Unlike the rapid decline in the capacity of Zn||Mn_2_O_3_ battery (capacity retention less than 50%), the capacity of Zn||O_Cu_-Mn_2_O_3_ battery still retains 88% of the initial capacity after 600 cycles at 1 Ag^−1^. In addition, Peng et al. prepared pristine V_6_O_13_ (p-VO) *via* electrodeposition and the self-assembly process, and then, oxygen-deficient V_6_O_13_ cathode (O_d_-VO) was obtained by annealing (Liao et al., 2020). Simulated results indicated that the introduced oxygen vacancy can reduce the Gibbs desorption free energy of O_d_-VO, which is more conducive to the desorption of Zn^2+^ than p-VO (shown in Figure 1B). The prepared O_d_-VO cathode has displayed roughly a capacity retention rate of 95% after 200 cycles at 0.2 Ag^−1^, which is significantly higher than p-VO cathode (collapsed within 180 cycles). Moreover, Kim et al. synthesized *in situ* growth of ZnMn_2_O_4_@C with Mn deficiency (Mn-d-ZMO@C) from the ZnO-MnO@C nanocomposite by solvent dry process and annealing methods (Islam et al., 2021). As shown in Figure 1C, ZnO-MnO@C transformed into Mn-d-ZMO@C *via* an aging process in electrolytes, which was along with the formation of Zn_4_(OH)_6_SO_4_·5H_2_O (ZBS) on the surface. Furthermore, Mn-d-ZMO@C and by-products realized reversible conversion by reacting with Zn^2+^ and Mn^2+^, respectively. The Zn/Mn-d-ZMO@C cell still maintained 84% of the highest capacity (98 mAh g^−1^) after 2000 cycles at 3 Ag^−1^. Thus, it can be seen that some vacancy optimization strategies reported recently have provided detailed analyses of the concentration and location distribution of introduced vacancies. However, more material systems need to be further studied to verify the universality of the optimization mechanism of this strategy.

### Substitution/Gap Doping

As reported earlier, the vacancy defects caused by doping modification have been confirmed stabilizing the crystal structure of cathode materials. Besides, the substitution doping of multivalent metal ions can effectively reduce the formation energy of cathode materials, which can effectively inhibit the collapse of crystal structure (Kim et al., 2021; Li et al., 2020). Ni et al. synthesized Mn-substituted zinc hexacyanoferrate materials (MZHCFs) using a simple precipitation method (Ni et al., 2021). The substitution of Mn ions in the N-bonded sites can restrain the cubic-rhombohedral phase transition and the dissolution of active materials in electrolytes, resulting in improving the structural stability. As shown in Figure 1D, the MZHCF (MZHCF-7) with Mn content of 7% retained 94% of the initial capacity (far more than 17% of ZnHCF) after 500 cycles at 0.25 Ag^−1^, displaying a significant synergistic optimization effect. In addition, the gap doping of heteroatoms (especially metals with similar ion radius) has been proved to effectively stabilize the phase transition structure and inhibit the dissolution of materials, which contributes to improving the reversibility of cathodic electrochemical reaction (Xu et al., 2021a; Chen et al., 2021). Moreover, Wang et al. obtained multivalent cobalt (Co^2+^, Co^3+^)-doped Mn_3_O_4_ nanosheets (Co-Mn_3_O_4_/CNA) based on carbon nanosheets array by electrodeposition on the basis of Co-MOF precursors prepared in water bath and annealing (Ji et al., 2021). Doped Co^2+^ in the interlayer of initial phase change products δ-MnO_2_ can play a supporting role due to strong adsorption energy (in Figure 1E). Meanwhile, doped Co^4+^ in the [MnO_6_] octahedral structure can improve the conductivity of Mn^4+^ and maintain a high specific capacity, which is owing to its low energy bandgap. In the subsequent charge–discharge process, cobalt with different valence states not only plays a supporting role in the phase change products but also can effectively inhibit the Jahn–Teller effect and promote the diffusion of ions. The prepared Co-Mn_3_O_4_/CNA cathode can still maintain 80% of the initial capacity after 1,100 cycles at 2 Ag^−1^. Nevertheless, the current research on doping modification has not further analyzed the influence of doping position and the proportion of different doping components on the stability of optimized materials. Furthermore, the similarities and differences of optimization mechanisms from different doping elements still need to be further discussed.

### Object Modification

**FIGURE 2:**
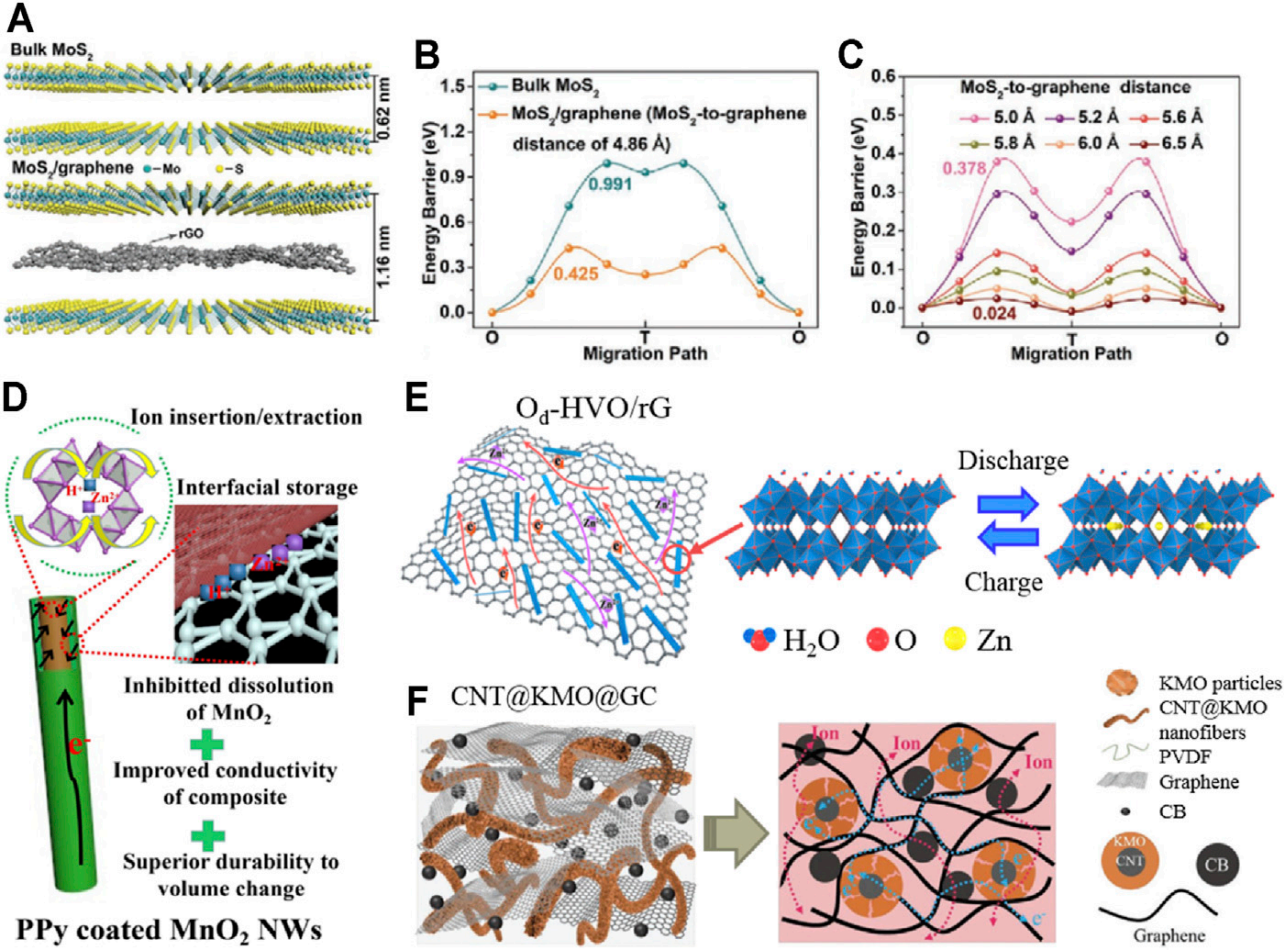
Structural modification and composite of cathode materials: **(A)** Crystal structures of bulk MoS_2_ and MoS_2_/graphene; **(B,C)** the corresponding migration energy barriers with the variation of the MoS_2_-to-graphene distance; **(D)** schematic illustration of freestanding CNT/MnO_2_-PPy; **(E)** schematic diagram of Zn^2+^ (de)intercalating mechanism in O_d_-HVO/rG; **(F)** illustration of electron/ion transport and ion diffusions across the electrodes of CNT@KMO@GC. Reproduced with permission (Zhang et al., 2020a; Li et al., 2021d; Huang et al., 2021; Wang et al., 2021).

The stability optimization strategy of cathode materials also includes object modification methods such as intercalation and surface coating. Moreover, object modification has been proved to effectively promote the reversibility of the reaction process and inhibit the dissolution of structures (Zhang et al., 2021). For layered cathode materials, the insertion of highly stable objects can promote the interlayer reversible transfer of Zn^2+^ (Liu et al., 2020b; He et al., 2021a; He et al., 2021b; Li et al., 2021c). Li et al. synthesized MoS_2_/graphene nanomaterials with a sandwich interlayer structure by solution stirring in an argon atmosphere at room temperature (Li et al., 2021d). Figures 2A–C show that reduced graphene oxide (rGO) was inserted between MoS_2_ layers, resulting in the significant expansion of the MoS_2_ layer spacing and the sharp decrease in the Zn^2+^ migration barrier. In addition, the stable flow structure alleviates the instability caused by interlayer stacking. The prepared cathode has a capacity retention rate of 88.2% after 1,800 cycles at 1 Ag^−1^, and its optimization effect is significantly outstanding compared with the previously reported transition metal sulfide cathode.

In addition, the surface coating belongs to the modification of the electrode/electrolyte interface, which is an effective strategy to inhibit dissolution and phase transformation of cathode materials (Gao et al., 2020). It has been confirmed that coating materials with high stability and conductivity can effectively improve the specific capacity and cycle stability of the cathode (Bin et al., 2021; Xu et al., 2021b; Ren et al., 2021; Xing et al., 2021). Yang et al. prepared an independent flexible membrane (CNT/MnO_2_-PPy) composed of carbon nanotubes and polypyrrole (PPy)-coated MnO_2_ nanowires through typical *in situ* reaction self-assembly and vacuum filtration (Zhang et al., 2020a). MnO_2_ nanowires coated with PPy (about 5 nm in thickness) are uniformly dispersed in highly conductive and interconnected carbon nanotube networks, improving the reaction kinetics and structural stability of the cathode (in Figure 2D). After 1,000 cycles at 1 Ag^−1^, the optimized electrode still maintained 87.4% of the initial capacity. Nevertheless, the range of structural modification materials used at present is limited, and the related synthesis processes still do not meet the needs of economic efficiency. Then, there are still some challenges in practical application.

### Combination Engineering

The adjunction of materials with a high stability structure for combination is also an exploration direction to improve the stability of cathode (Zhang et al., 2020b; Shan et al., 2021). The optimization strategy of combination engineering usually includes carbon-based materials, which can improve the electron transmission efficiency and structural stability of materials (Yang et al., 2021; Zeng et al., 2021). Hou et al. synthesized a 3D reticular graphene-based hydrated vanadium dioxide composite (O_d_-HVO/rG) with abundant oxygen vacancies using the solvothermal method (Huang et al., 2021). The research confirmed that oxygen vacancy defects can provide more active sites and promote the reversibility of the reaction, while the highly conductive and robust rG sponge can promote electron migration and reduce the accumulation of O_d_-HVO to improve the conductivity and structural stability, as shown in Figure 2E. Compared with HVO (capacity retention of 86.5%) and Od-HVO (capacity retention of 93.6%), the O_d_-HVO/rG cathode expressed scarcely any attenuation after 750 cycles at 5 Ag^−1^. Moreover, Li et al. obtained a cathode material (CNT@KMO@GC) composed of graphene (G), carbon black (CB), and K-sodium manganite (K_x_MnO_2_·yH_2_O, KMO) based on core–shell carbon nanotube (CNT) by hydrothermal and solution treatment (Wang et al., 2021). In Figure 2F, KMO provides the main charge storage due to the interlayer intercalation of K^+^ and H_2_O; CNT provides a conductive framework for the loaded KMO owing to high conductivity and structural stability; G and CB provide the conductive network to reduce the accumulation of active substances. The prepared cathode has a capacity retention rate of 65.2% after 10,000 cycles at 5 Ag^−1^, which is significantly higher than KMO (39.1% of the initial capacity) and CNT@KMO (51.5% of initial capacity). However, the influence of the composite ratio on stability optimization has not been deeply analyzed, and the composite research of non-carbon matrix materials needs to be further explored. Chen et al. revealed the performance attenuation mechanism of MnO_2_-based AZIBs by contrasting with different polymorphs and found that the low manganese dissolution of R-MnO_2_ inhibits the degradation of performance (Liao et al., 2022). Therefore, the reasonable composite design of MnO_2_ polymorphs with high initial capacity and R-MnO_2_ may have certain advantages in capacity and stability compared with single crystal form, which provides a direction for the next optimization.

## Summary and Perspectives

In summary, the progress of cathode stability optimization for aqueous zinc ion batteries has been reviewed; the main of which can be divided into four aspects, including the introduction of vacancy, substitution/gap doping, object modification, and combination engineering. Thus, cathode stability optimization strategies can be designed from three aspects: inhibiting material dissolution, improving reaction reversibility, and enhancing structural stability.

However, there are several aspects to be further researched in the aforementioned optimization schemes of cathode materials. For quantitative analysis, most of the doping and composite research studies lack exploring the relationship between concentration/location and the optimization degree of stability. For universality analysis, material systems introduced into optimization research are still limited. For practical application, some synthetic processes, such as surface coating, still need to meet the demands of the economy, efficiency, and safety. In addition, the realization of the most stable cathode performance needs to eliminate the factors that reduce the reversibility according to the reaction mechanism of materials, such as inhibiting the irreversible dissolution of materials and the formation of inert by-products. Therefore, these fields to be explored can be the focuses of stability optimization in the future.
